# Supplementary figures and images for: Shift in vacuolar to cytosolic regime of infecting Salmonella from a dual proteome perspective
Source: PLoS Pathog. 2023 Aug 3;19(8):e1011183. doi: 10.1371/journal.ppat.1011183 (PMC10426988; doi:10.1371/journal.ppat.1011183)

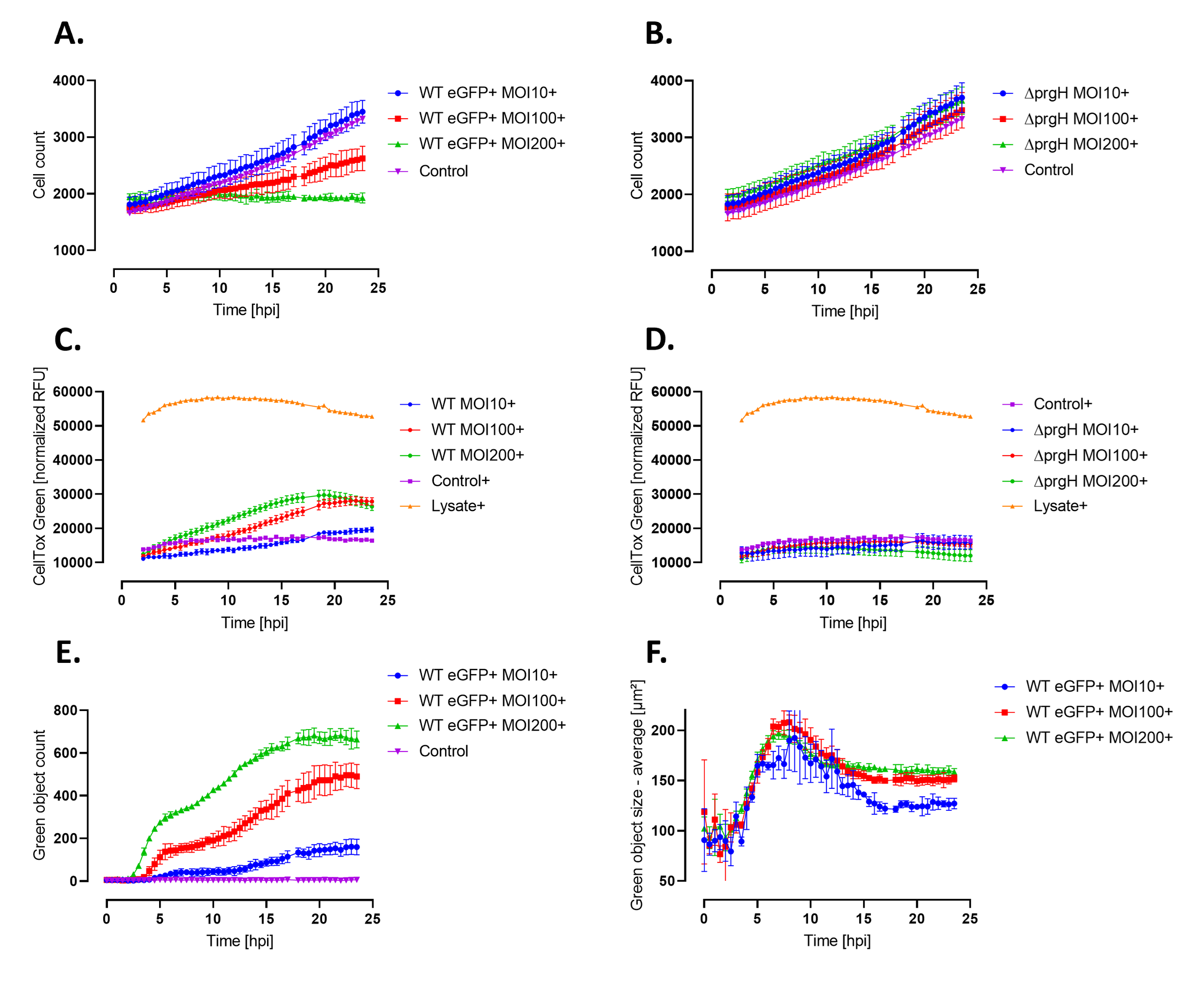

Supplement: S1 Fig — HeLa cells (1,875 x 10^4^ HeLa cells) were non-infected or infected with S. Typhimurium (WT or ΔprgH SL1344) at MOIs 10 (blue), 100 (red) or 200 (green) in quadruplicate and cell count and cytotoxicity (or GFP fluorescence) measured in a multi-well optical plate reader in real-time. Label-free cell counting (10x objective) of HeLa cell cultures infected with WT Salmonella (A) or non-invasive ΔprgH mutant Salmonella (B). Monitoring cell death in real-time using the CellTox Green Cytotoxicity Assay (C-D). Using a constitutively eGFP-expressing strain of SL1344 [45] (WT eGFP) green objects were counted and their average size (μm2) determined (F). Due to technical measurement errors, the 17.5 and 18 hpi recordings were omitted. (TIF) [file ppat.1011183.s001.tif]

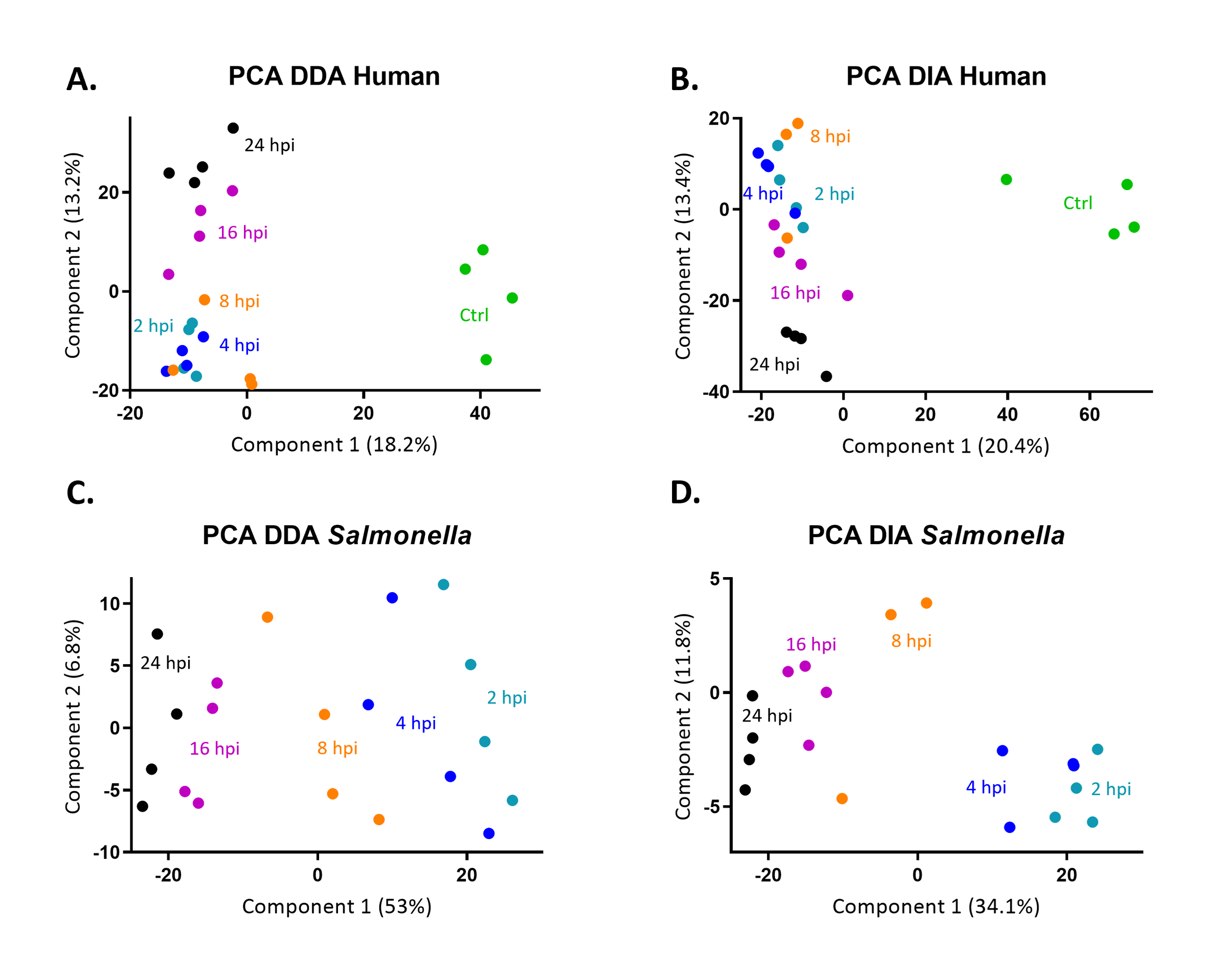

Supplement: S2 Fig — Individual DDA (A-C) and DIA (B-D) PCA plots for Salmonella (C-D) as well as HeLa (A-B) over the time-course of infection. Green, cyan, blue, orange, purple and black circles represent control (Ctrl), 2, 4, 8, 16 and 24 hpi samples, respectively. (TIF) [file ppat.1011183.s002.tif]

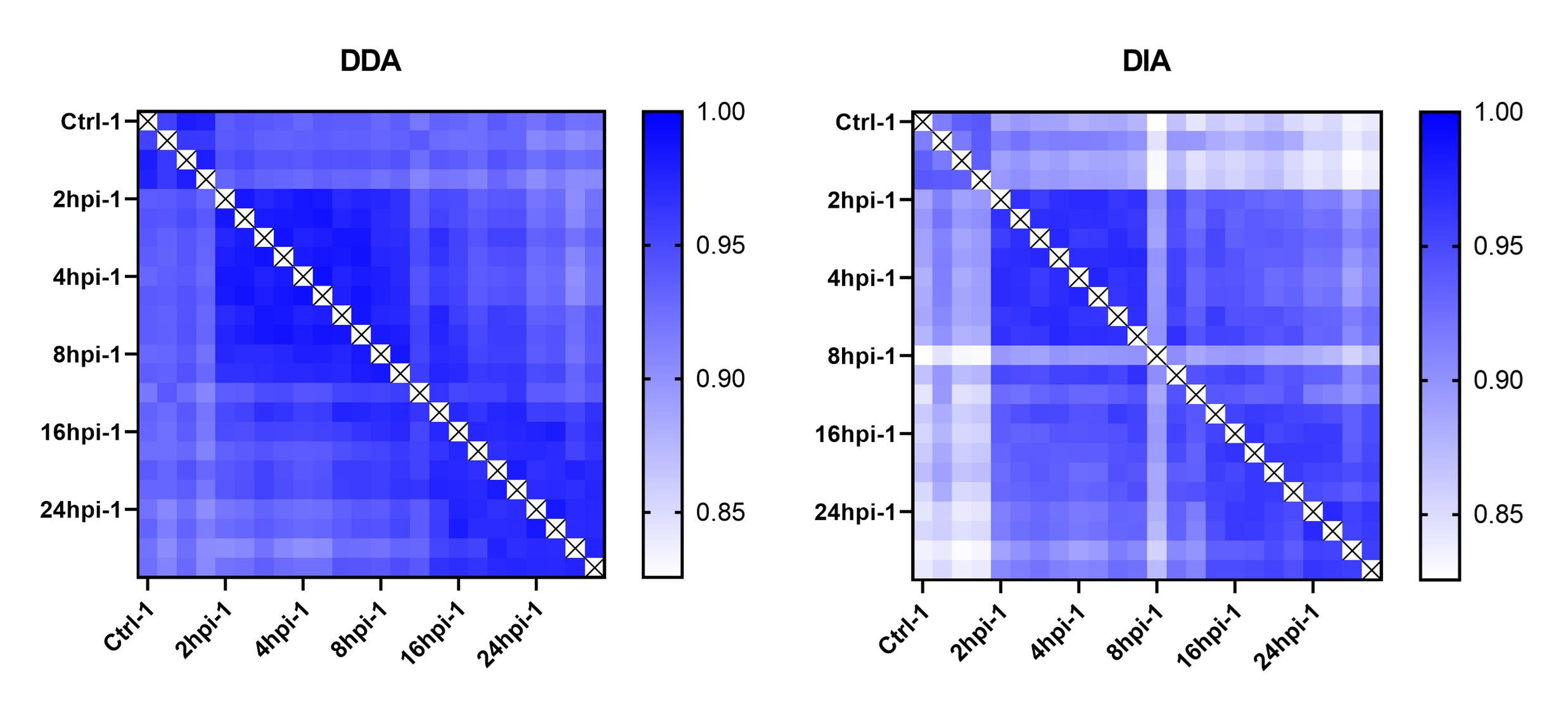

Supplement: S3 Fig — Ctrl HeLa, 2 hpi, 4 hpi, 8 hpi, 16 hpi, and 24 hpi. (TIF) [file ppat.1011183.s003.tif]

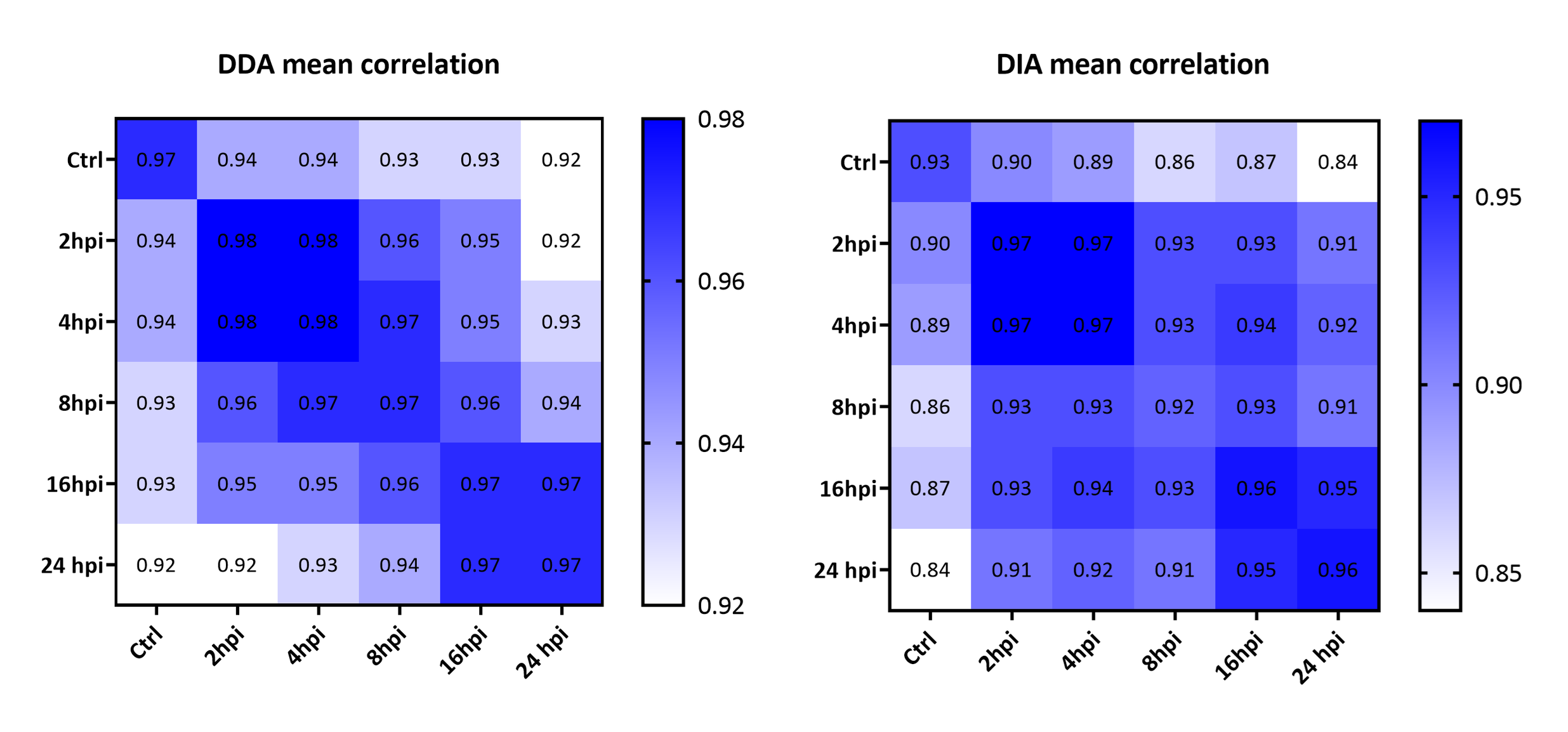

Supplement: S4 Fig — Ctrl HeLa, 2 hpi, 4 hpi, 8 hpi, 16 hpi, and 24 hpi. (TIF) [file ppat.1011183.s004.tif]

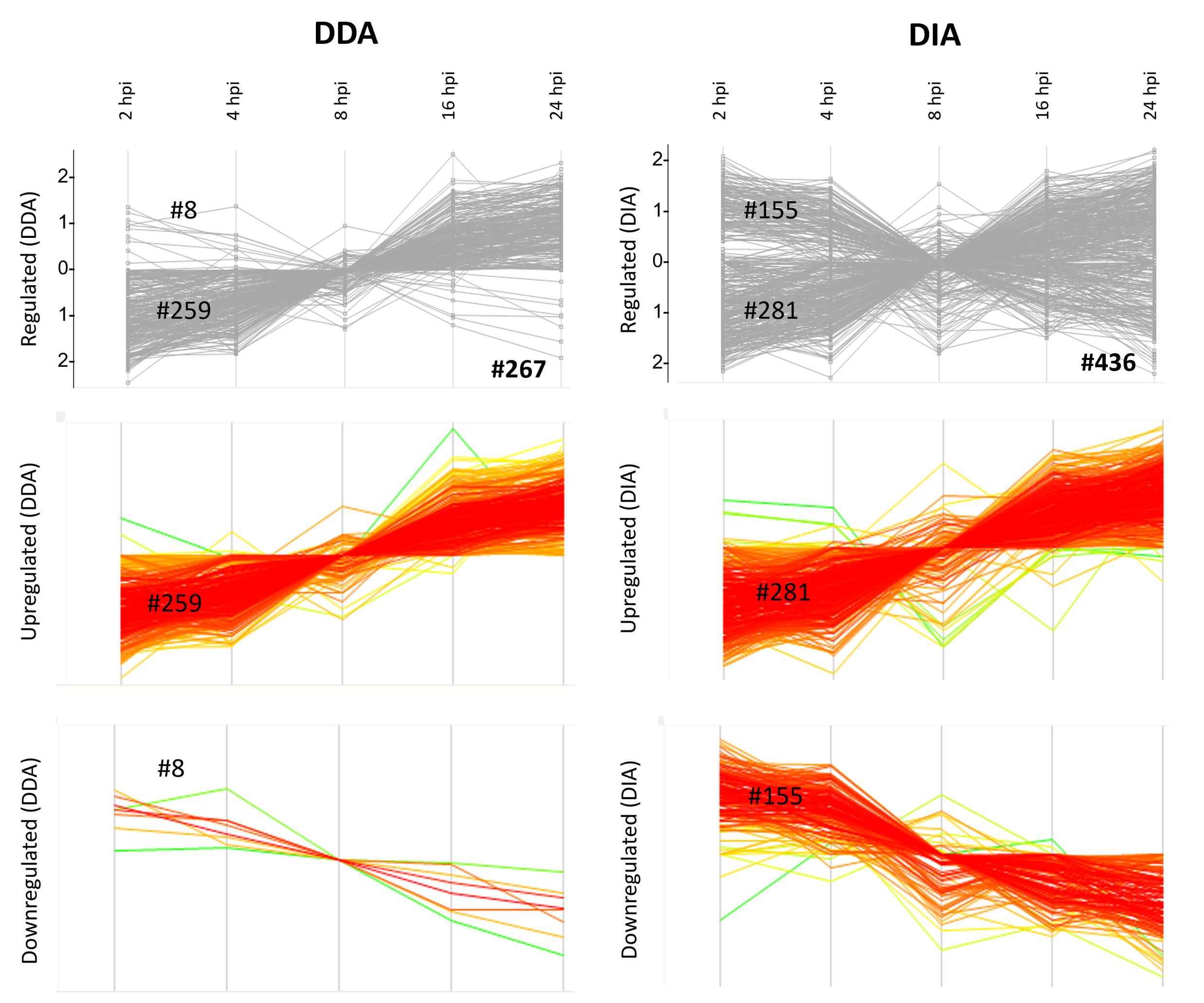

Supplement: S5 Fig — Significant regulation (FDR ≤ 0.01) was determined across the 5 timepoints post-infection using multiple t-testing and corresponding normalized averaged z-scores of S. Typhimurium DDA (left panels) and DIA data (right panels) plotted, with corresponding members of the two main clusters (i.e., up- and downregulated cluster) shown and its corresponding number of regulated S. Typhimurium protein groups indicated. (TIF) [file ppat.1011183.s005.tif]

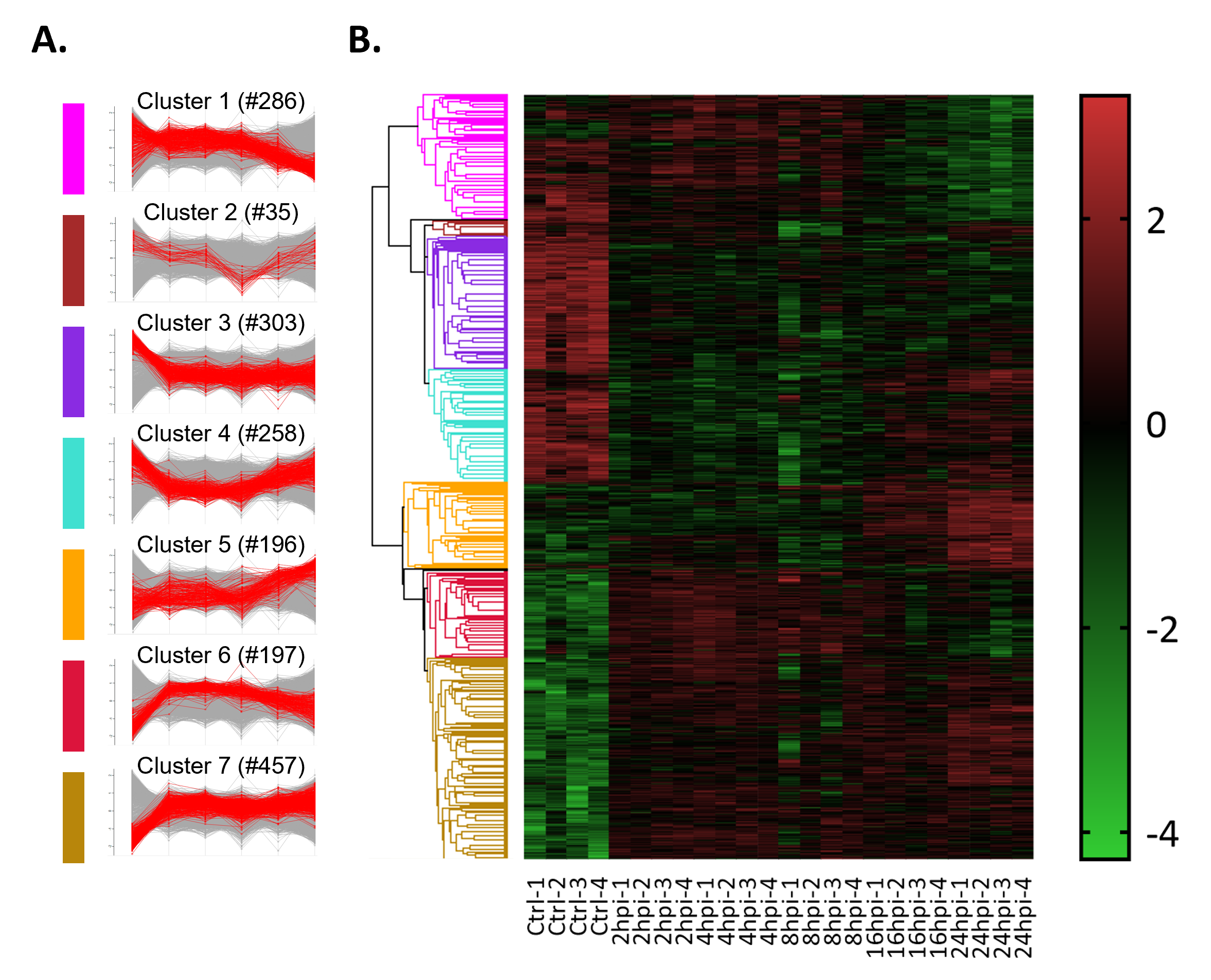

Supplement: S6 Fig — Regulation was determined across the 5 timepoints post-infection and control cells using t-testing for normalized DIA data. The intensities of significantly (FDR ≤ 0.01) regulated human proteins (#1,742) from the DIA analysis are shown. The averaged expression values of corresponding members of the 7 main clusters after ANOVA are shown as profile plots (A) and as a heat map (B), with the corresponding number of regulated protein groups indicated per cluster (see also S5 Table). In panel B, green indicates low intensities while red indicates high intensities. (TIF) [file ppat.1011183.s006.tif]

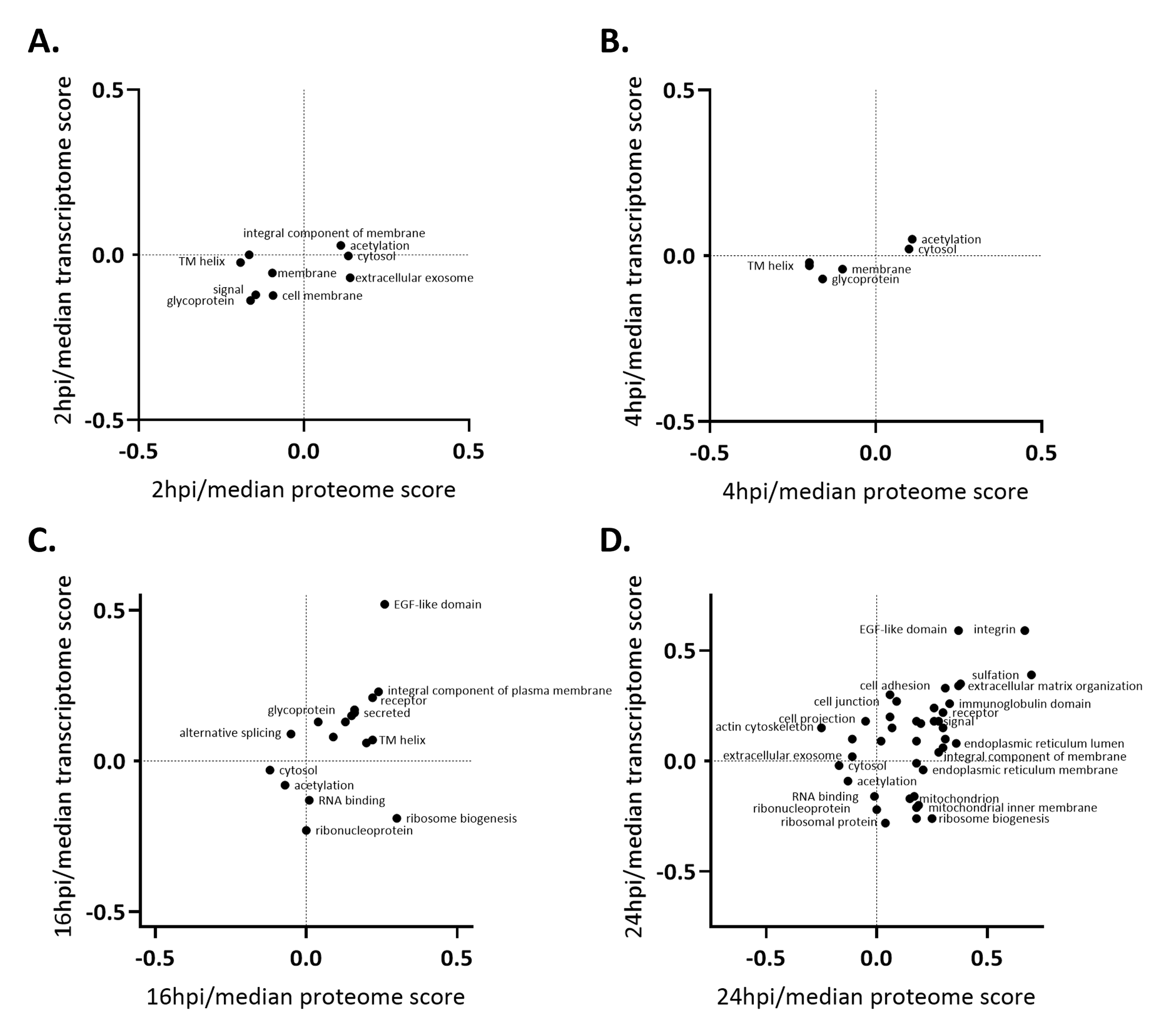

Supplement: S7 Fig — The 2D-annotation enrichment scores for representative GO biological processes (GOBP), GO cellular component (GOCC), GO molecular function (GOMF) and keywords were plotted for the proteome (DIA data) and transcriptome expression changes (GEO series accession number project GSE60144, [41]) at 2, 4, 16 and 24 hpi (no 2D enriched terms were observed at 8 hpi). The fold change of log2 transformed means of corresponding LFQ and RPKM values were plotted and only corrected p values ≤0.01 were considered. (TIF) [file ppat.1011183.s007.tif]

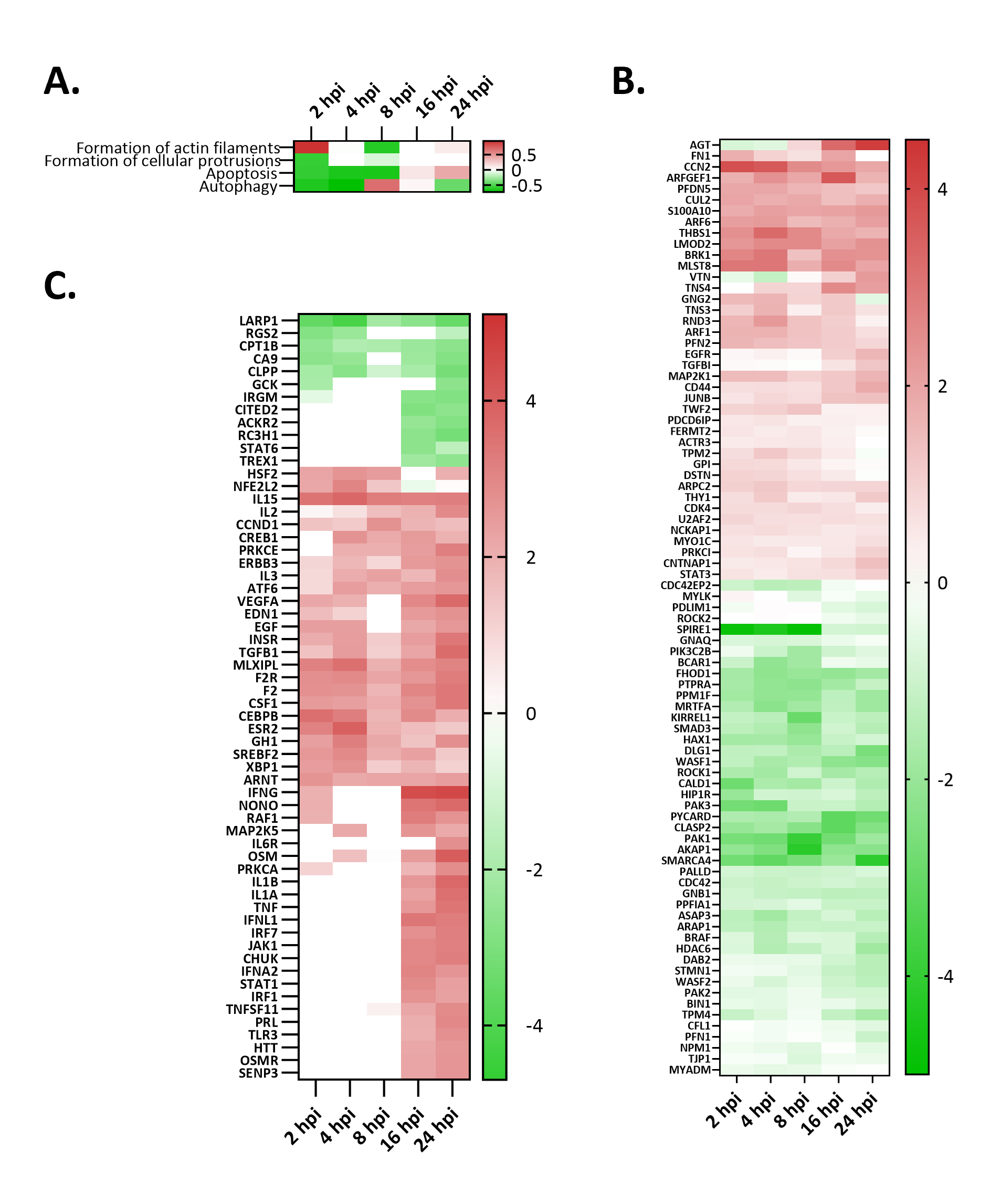

Supplement: S8 Fig — (A) Heatmap visualization (IPA activation z-score) of selected IPA functions regulated over the course of the infection. (B) Overview of regulated genes (#85) (log FC) contributing to the IPA function “formation of actin filaments” (see also S5 Table). (C) Overview of activated and inhibited upstream regulators. Heatmap representation of 60 predicted proteinaceous (activated or inhibited for at least one of the timepoints post-infection analysed) upstream regulators (p-value ≤ 0.01), colour coded according to the IPA activation z-score. (TIF) [file ppat.1011183.s008.tif]
