# Supplementary material for: Shift in vacuolar to cytosolic regime of infecting Salmonella from a dual proteome perspective
Source: PLoS Pathog. 2023 Aug 3;19(8):e1011183. doi: 10.1371/journal.ppat.1011183 (PMC10426988; doi:10.1371/journal.ppat.1011183)
Supplement: Attachment — Submitted filename: Response to Reviewers.pdf [file ppat.1011183.s017.pdf]

Response to Reviewers comments Part I - Summary

Reviewer #1:

R1 - In the manuscript entitled “Shift in vacuolar to cytosolic regime of infecting Salmonella from a dual proteome perspective” by Fels U. et al. attempted to dissect translated proteins both in host cell (HeLa cell) and Salmonella Typhimurium over course of bacterial infection by a new proteomics approach called “dual proteome profiling”. With using the approach, they found DIA mode is better than DDA one to detect low expressed peptides in Salmonella and could show trajectory of their expressions during infection, also patterns of some expressed proteins were consistent with previous reports. New proteomic approach that the authors established previously is potentially useful to analyzed proteins of both host and bacteria without enrichment, however, this version of manuscript has some serious issues. The proteomic approach remains incomplete, and results obtained by the analysis look not bringing new findings in this manuscript. Here, I would like to list up more detail. Addressing the questions and changing manuscript would be better suited for the publication.

We would like to thank the reviewer for acknowledging the potential of our dual proteome profiling strategy to study host/pathogen systems. We addressed the concerns raised in this general comment, in our below answers to the specific detailed comments made by the reviewer.

Reviewer #2: Novelty:

R2 - this is well-studied system so the novelty lies in the quality and depth of the analysis, and the conclusions that these deep measurements allow them to draw
Strengths:
- very strong technical approach to studying an important host/pathogen system
- very well executed
- appropriate infection model (MOI, time course, gentimycin addition, etc)
- some appropriate analyses of their data and comparisons to others

We would like to thank the reviewer for her/his kind appreciation of our work and acknowledging the methodological and technical advances of the dual proteome profiling approach applied to a well-studied system.

R3 - Areas to improve:

- I would suggest some more comparisons to or mentions of some of the other proteomic studies that have been done on this system. I don't care so much about comparisons to transcriptomic studies - those typically aren't so relevant for understanding what happens at the proteome level anyway.

By making use of a well-studied system and viewing the absence of detailed functional follow-up studies, we focused on the methodological advance as the main focus of the presented research. However, the comment of the reviewer is very valid, and we now included more mentioning of other proteomic studies besides the relevant studies already referenced (e.g., (1-8)). While we point out overlapping findings, we believe that detailed data comparisons are complicated viewing the multifactorial complexity of host-pathogen interactions as also discussed in (9) (e.g., (bacterial) cultivation, host susceptibility (e.g. the varying phenotypic responses of different HeLa cell lines to Salmonella infection as reported

in (10)), differences in infection conditions (MOIs) or experimental setup used (e.g., prior fractionation of cytoplasm and nucleus in (1)), especially when considering the integrative proteomic perspective of our study. Nonetheless, besides pointing to established findings confirming the validity of the dual proteomics profiling strategy presented, we now further emphasized the novel findings of our dataset (see also below comments).

R4 - one of the key features of Salmonella's infection system is its ability to establish the intracellular niche that it depends on to be able to replicate within the host cell. There is some analysis related to potential factors involved in surviving in the cytosol (lines 304 and onward) but mostly Salmonella needs to take over internal membrane systems (the Salmonella-containing vacuole and filaments). As I was reading the manuscript, I was hoping to learn something about how there might be changes in proteins involved in those processes. The data presented in Fig. 5 starts to go in that direction but only from the Salmonella side of the equation.

*Thank you for this important comment. It is indeed so that creation of an extensive membrane network within its host lies at the basis of Salmonella's infection strategy and that intracellular proliferation of Salmonella is directly correlated with the ability to transform the host cell endosomal system into a complex tubular network highly dependent on the translocation of effector proteins. Consequently, an in depth understanding of the proteins implicated in those processes throughout the infection is of high importance. To obtain more detailed insights into expression changes of the proteome of internal membrane systems, we turned to a study reporting on a comparative proteome analysis of S. Typhimurium infected HeLa cells enabling the identification of host proteins associated with Salmonella-modified membranes (SMM) (11). More specifically, SMM proteins were shown to be enriched for transport and membrane linker proteins, GTPases, cytoskeleton proteins as well as proteins implicated in clathrin and coatomer-mediated transport (11). From the high-confidence set of SMM host proteins reported (247 proteins), 213 proteins (86%) were also identified in our DIA analyses. Interestingly, of these, 84 SMM proteins (39%) were found to be significantly regulated after multiple sample ANOVA testing (FDR ≤ 0.01 and an S0 of 0.1) (see two added columns entitled 'SMM proteome (PMID: 25348832)' and 'ANOVA regulated SMM protein' in the revised version of **Supplementary Table S2C**). By itself, regulation of SMM protein expression over the time-course of infection was found to be highly statistically significant as determined by a chi-square test of independency (p < 0.01). Regulated SMM proteins included among others the transport protein Phosphate carrier protein MPCP, the Translational endoplasmic reticulum ATPase (VCP), the ATP synthases F(0) complex subunits 1 and 0, AP2A1, AP2B1 and TMED9 implicated in clathrin/vesicle-mediated transport, and the cytoskeleton and membrane linker proteins Ezrin, Plastin-3, Myosin I, the Arp2/3 complex and Catenin alpha-1 and several GTPases (including the over 10-fold upregulation of the Ras-related protein Ral-A identified in our study) (new **Supplementary Table S4**). In line, 1D annotation enrichment analysis showed significantly reduced protein abundancies of SMM proteins in the control setup, pointing to an overall general increase in SMM proteins upon Salmonella infection (p-value < 0.01). While validating the regulated SMM host targets identified in our study falls outside the scope of the current study, it is noteworthy that these regulated targets hold several components of host pathways previously shown to be implicated in formation of SCVs or SIFs (e.g., endosomal and recycling pathways). These regulated targets include amongst others the small Rab GTPases Rab7a (12), Rap-1b (13) and Rab10 (11) – all with known fundamental roles in cellular membrane dynamics besides representing hub targets of bacterial effector proteins (14), besides the observed regulations of the AP-2 complex subunit β (AP2B1) (13), the F-actin binding protein utrophin (11), and the cytoskeleton(-linked) proteins desmoplakin (15) and Filamin (16).*

Overall, this clearly demonstrates that we identified a multitude of infection-relevant host proteins from the SMM to be regulated over the time course of infection, thereby representing interesting targets for further follow-up studies. A new supplementary Table including 29 ANOVA regulated SMM proteins (from the 84 regulated SMM proteins identified in total) signifying GTPases, or proteins implicated in transport or cytoskeleton and membrane organizations (selected form keywords) is now included as ***Supplementary Table S4*** *and the related findings discussed in the revised version of the manuscript. As such, our work reports for the first time the regulation of protein expression of host proteins associated with Salmonella-modified membranes in the context of infection, including some characterized targets implicated in formation of SCVs or SIFs.*

Response to Reviewers comments Part II - Major Issues: Key Experiments Required for Acceptance

Reviewer #1:

R5 - The new approach "dual proteome profiling" without a priori bacteria enrichment in this study sounds attractive but remains incomplete. Salmonella proteome contains around 4,500 proteins but this approach detected only less than 900. Previous reported proteomic research demonstrated detection of Salmonella proteins around 1,800 with enrichment infected cells (Li et al, mSystems, 2019; RMID:30984873), suggesting "dual proteome profiling" is difficult to be taken the place of previous approach without further modification.

In our study we detected nearly 1000 (i.e., 991) S. Typhimurium proteins representing 21% of the annotated, and 24% of the expressed S. Typhimurium proteome (i.e., when considering that by means of ribosome profiling, we previously found that 4135 genes (~90%) were found to be expressed across a multitude of growth conditions, thus likely even an underestimation (17)).

As indicated in our original text (i.e., 'Bacterial proteome sampling in dual proteome profiling nevertheless remains less comprehensive as compared to previously reported protein identifications making use of pure Salmonella cultures or of Salmonella isolated from infected epithelial host cells. As we found a typically 2-fold increase in the number of S. Typhimurium proteins identified and quantified (around ~1,500 to 1,600) using similar MS instrumentation in DDA mode.'), the reviewer indeed correctly points out the significant higher number of Salmonella proteins identified (~1,800) in the Li et al study making use of enriched Salmonella from infected cells (3). In this context, it is however important to note that while we analysed unfractionated samples using 1D LC-MS/MS, Li et al (3) made use of extensive fractionation by means of 1D SDS-PAGE separation and slicing of the gel prior trypsinization into 36 (!) gel pieces per biological replicate sample analysed, a strategy which overall resulted in an unprecedented high number of identified Salmonella proteins in the context of infection. Offline sample fractionation in combination with DIA can thus provide a useful asset to improve our DIA identification rates further, especially in the case of complex proteome mixes with components of low abundance (18). So when increasing LC-MS/MS run time by, for instance, offline pre-fractionations or the use of parallel DIA runs with narrow m/z windows in consecutive m/z ranges as demonstrated in the EncyclopeDIA workflow (19), a significant increase in the identification of Salmonella proteins is surely expected in case of dual proteome profiling, meaning that this does not represent an inherent shortcoming of dual proteome profiling strategy per se. We now additionally indicated this important point in the text.

Of additional importance, the dual proteome profiling strategy presented leaves the isolated proteome unperturbed due to the instant nature of the proteome isolation (i.e., not requiring the rather lengthy and also potentially biased enrichment procedure needed for the isolation of intracellular bacteria) while nonetheless (already) providing a significant Salmonella proteome coverage. Moreover, DIA dual proteome profiling provides a very high quantification sensitivity, which in our study permitted the reliable capture of over 100 regulated bacterial proteins over the time course of infection, a number that generally exceeds the numbers of regulated genes reported in other studies, even when making use of enriched Salmonella (6), thereby providing convincing leads for further follow-up studies.

R6 - Human sample contains a control (noninfected control), but Salmonella sample doesn't contain any proper control. Expression of bacterial proteins are quickly changed by response to environment. I think "2 hpi" is too late to see early time responses and compare with the responses at later time points, thus "0 hpi" sample for bacterial side should be needed. Furthermore, in Salmonella proteomic results, I think new findings seem not to be presented in the manuscript.

As it is highly debatable what serves a proper Salmonella control, also viewing the quicky changing environment as indicated by the reviewer (i.e., from rich growth media, to washing in HBSS buffer and resuspension in DMEM medium, followed by host internalization etc.), and in line with the relative comparisons of Salmonella protein expression at various time-points post-infection already reported in literature (6), we opted to make use of a Salmonella 2 hpi reference. Consequently, as correctly pointed out by the reviewer, initial bacterial adaptations to the host might indeed have been missed, as acknowledged by us in the original manuscript. Viewing the scarce bacterial load at earlier time points and the higher variability of quantification data observed for very low abundance species given the interference with random noise (18), no time-points prior 2 hpi were considered. Further, by comparing expression changes in host 'adapted' Salmonella, expression changes more accurately reflect expression changes throughout the infection process itself (e.g., expression changes due to changes in subcellular localization of Salmonella within the host). Representative of this, is that upregulation of e.g., phosphate utilization, previously shown to be highly induced in the Salmonella proteome upon medium switching occurring prior to bacterial internalization (20), was indeed not observed in our study.

R7 - The author should prepare graphs in figures representing that they want to say. In some places, authors take us to see supplementary tables, but such tables are just lists of protein expression data. For example, "….and unique number of peptides identified (Supplementary Tables S1A-B)" on line 158 of the manuscript, I couldn't figure out "unique number of peptides" on the tables. "…significant upregulation of metal ion transporters and… (Supplementary Table S2D)" on line 291, representing by graphs should be better. In addition of that, some figures in the manuscript need to be modified. Fig. 1 should add PCA of Salmonella in the over course of infection as well as host cells. Individual protein expression patterns regulated by the regulators (e.g., hilA, phoPQ, or others) in Fig. 5 should be shown. I also don't know why Fig. 6 is involved in Discussion. It should be moved to Result section.

We thank the reviewer for his/her suggestions and respected remarks. Since at certain occasions numerical data was indeed not included and only indirectly referred to in the Supplementary Tables, we now included more detailed information in the relevant text, including among others the info on the unique number of peptides identified. All the significantly upregulated (cluster up) metal ion transporters and uptake systems identified have been specified in the text (i.e., ZnuA (zinc), IroBN, EntBF, SitAB, Fur, Mrp, FepAB, CirA

(iron) and SitAB (manganese)). Maximum fold-upregulation at 24 hpi have now additionally been mentioned.

Related to including extra graphs, and while we originally opted to include easy interpretable figures with high data content (e.g. original figures 4 and 5 showing respectively heatmaps of enriched human annotation terms and bacterial regulon scores across all conditions analysed) to provide a more global picture of the expression changes observed, thereby not cherry-picking from the wealth of data also viewing the lack of functional validation, we nonetheless agree that additional graphs may facilitate data interpretation. We now included extra graphs or details for the data when discussed in the text, next to moving supplementary fingers to the main text (see below comments). For all regulated proteins however, patterns of regulation can readily be inferred from the heatmap (now ***Supplementary Figure S6****) and corresponding cluster annotations in the Supplementary files.*

*Moreover, a new Supplementary Figure (****Supplementary Figure 2****) has been included which shows the individual DDA and DIA PCA plots for Salmonella as well as HeLa over the time-course of infection.*

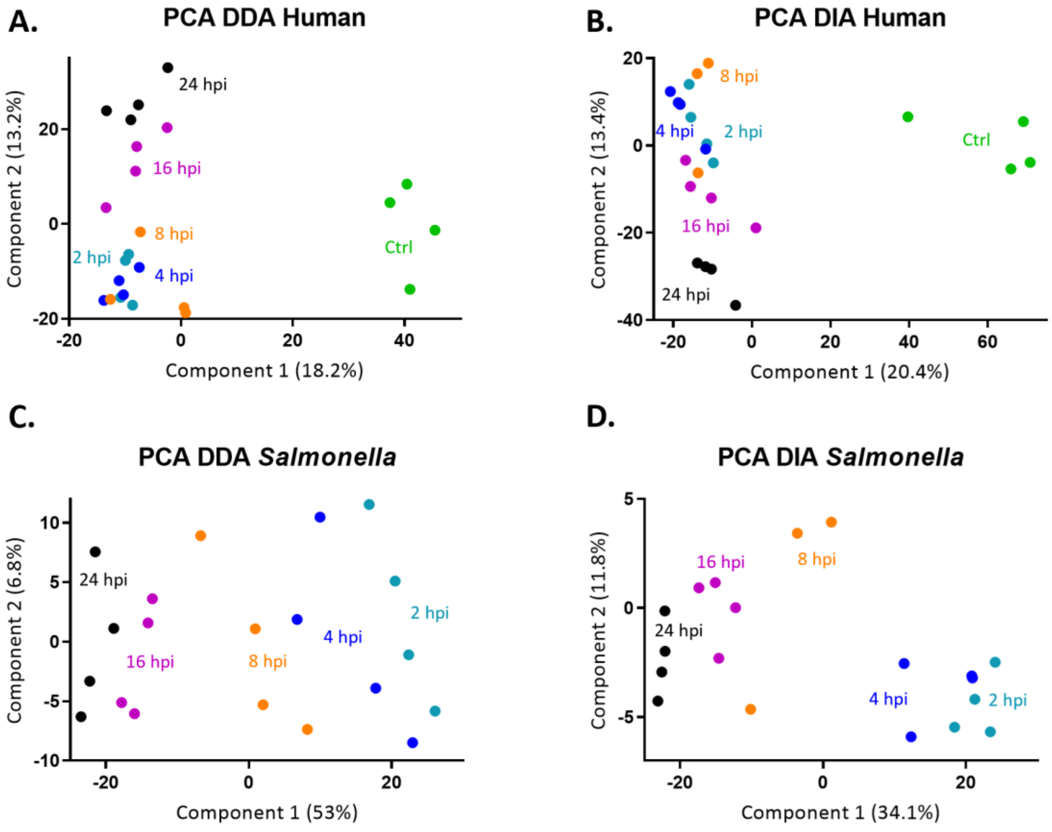

Figure S2| Variability of DDA and DIA replicate samples represented in PCA plots. *Individual DDA* ***(A-C)*** *and DIA* ***(B-D)*** *PCA plots for Salmonella* ***(C-D)*** *as well as HeLa* ***(A-B)*** *over the time-course of infection. Green, cyan, blue, orange, purple and black circles represent control (Ctrl), 2, 4, 8, 16 and 24 hpi samples, respectively.*

*Here, from the host perspective, the PCA clearly distinguishes non-infected versus infected host cells (component 1, 18.2% and 20.4% for DDA and DIA data, respectively), besides revealing a temporal response (component 2, 13.2% and 13.4%) (****Fig. S2A-B****). This temporal*

*response is also reflected in the recordings of the pathogen (component 1, 53% and 34.1%) (**Fig. S2A-B**) .*

Individual protein expression patterns regulated by the regulators (e.g., hilA, phoPQ, or others) have been included as an extra panel (panel B) in Figure 5.

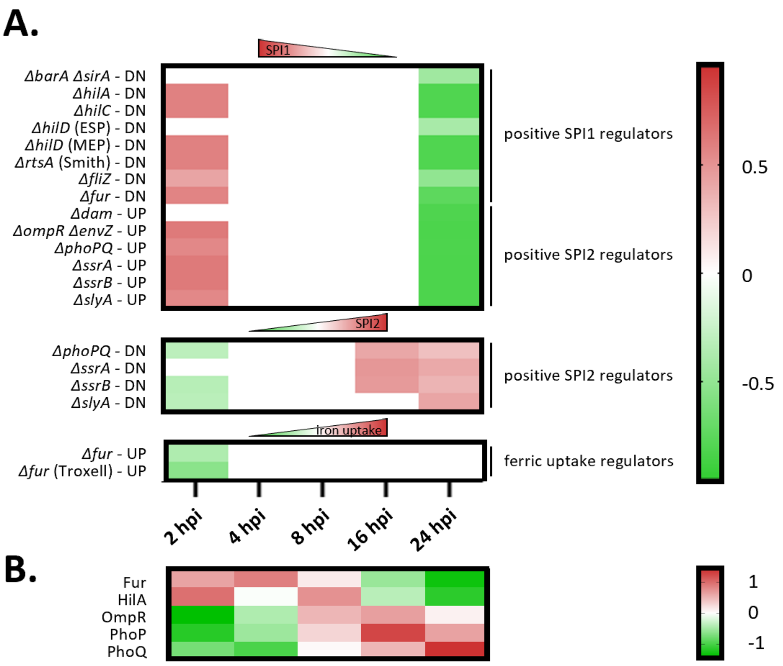

Figure 5| Heatmaps of significantly regulated (FDR ≤ 0.05) normalized S. Typhimurium regulon enrichment scores** and **expression changes of their identified regulators across all infection conditions assayed by data-independent acquisition (DIA). **(A)** *Using a recently reported compilation of highly curated regulon gene targets identified by means of microarray or NGS-based gene expression studies (2), enrichment of specific regulons was determined using the 1D annotation enrichment algorithm embedded in the Perseus software suite. P-values were corrected for multiple hypotheses testing using the Benjamini and Hochberg false discovery rate. Enriched annotation terms (FDR ≤ 0.05) in the categories are shown as heatmaps and colour coded according to the 1D enrichment scores calculated (21), with red and green colouring indicating enriched and depleted annotations, respectively. ESP (early stationary growth phase), MEP (mid exponential phase), upregulated (UP) and downregulated (DN) genes as reported in (2).* **(B)** *Heatmap representation of significantly regulated S. Tyhimurium regulators (FDR ≤ 0.01, corresponding normalized averaged z-scores) from the DIA analysis and corresponding to the regulated regulons shown in A are shown. SsrB was also identified, but expression was not significantly regulated.*

*Finally, Since the original Figure 6 mainly supported some of the more general findings discussed in these context, we preferred to reference the figure in the discussion, though moved it to the Supplementary Figures (i.e. **Supplementary Figure S7**).*

R8 - Some biological experimental designs are not good. Authors show intracellular bacteria cfu (in well? per mL?) and cytotoxicity induced by infection with Salmonella in Fig. S1, it should include results at later time points (16 hpi and 24 hpi). Intracellular cfu of a prgH mutant stain should be shown in Fig. S1A as well. In the LDH cytotoxicity assays, “Target Cell Maximum LDH Release Control” is required (see the instruction), otherwise it can’t be rule out that possibility of “non-Infected” control cells are also dying.

This info was indeed only explicitly specified in the methods section. As such, in the original version of Supplementary Figure S1 the bacterial enumeration by CFU counting (and thus intracellular bacteria) was presented per 18,750 HeLa cells as counted per well, and thus indeed per 96-well.

To give a more detailed overview of the intracellular growth and recording of Salmonella, besides monitoring of cytotoxicity in the infected HeLa model system under study, real-time cell count and confluency measurements (as proxy of cell viability) as well as fluorescence measurements to monitor infection progression making use of the GFP-expressing Salmonella SL1344 strain JVS-3858 (22) (as inferred from the green object counts) was performed in real-time. We now replaced the original Supplementary Figure 1 with the below results. A non-invasive prgH mutant strain was also included as control and related methods have been introduced in the relevant sections.

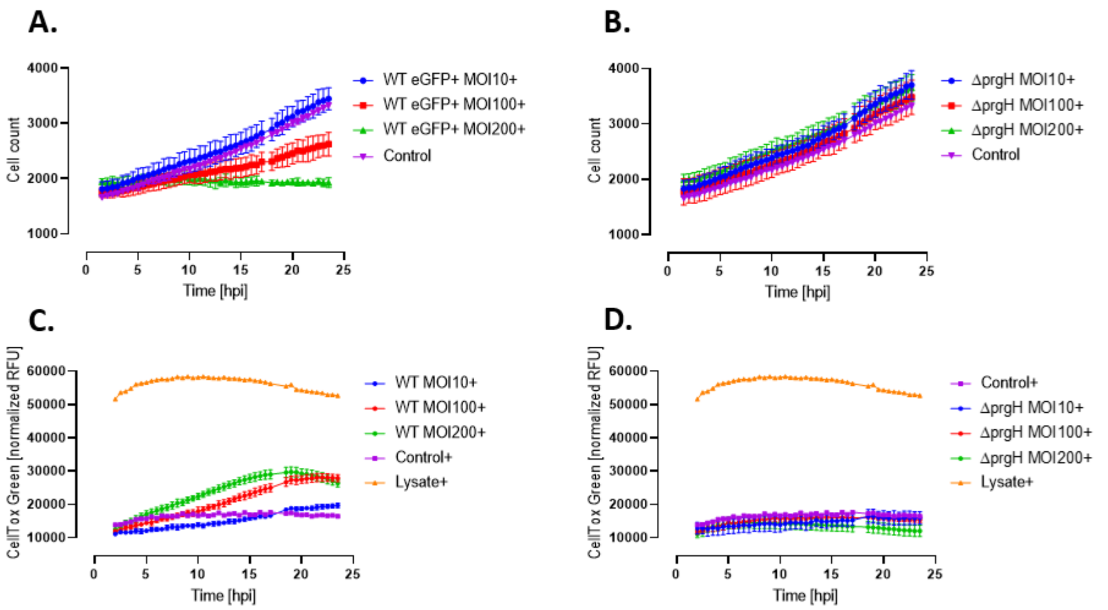

Figure S1| *Characterization of epithelial Salmonella infection model. HeLa cells (1,875 x 10^4 HeLa cells) were not infected or infected with S. Typhimurium (WT or ΔprgH SL1344) at MOIs 10 (blue), 100 (red) or 200 (green) and cell count and cytotoxicity measured in real-time. Label-free cell counting (10x objective) of HeLa cell cultures infected with WT Salmonella* ***(A)*** *or with non-invasive ΔprgH mutant Salmonella* ***(B)****. Cell death monitoring in real-time using the CellTox™ Green Cytotoxicity Assay* ***(C-D)****. Due to technical errors during measurement, the 17.5 and 18 hpi recordings were omitted.*

Reviewer #2: I do not feel that there are additional wet experiments required. Rather, the following bioinformatic analyses could enrich the manuscript:

R9 - comparison to other Salmonella proteomic studies - similarities/differences in classes of proteins expressed

We would like to refer to our answer addressing related comments ***R3*** *and* ***R4****.*

R10 - analysis of host membrane trafficking proteins altered in their study

We would like to refer to our answer addressing the previous related comment ***R4****.*

Reviewer #3:

R11 - The work seems to be well done and is clearly written. The authors present a more comprehensive proteomics analyses and identify more proteins than published before, however, my main problem is that I did not see what the new findings are, or they were not presented well. All results seem to be confirmatory (references are given) and I am missing that a specific, new result is highlighted. Thus, I recommend to better define the novelty of the results obtained by this dual proteomics approach and to better discuss how the host cell and the pathogen interact, react to each other.

We would like to thank the reviewer for her/his kind appreciation of our work. By making use of a well-studied system and viewing the absence of detailed functional follow-up studies, we focused on the methodological advance as the main focus of the presented research, but now included and highlighted some new results, such as reporting for the first time the regulation of protein expression of host proteins associated with Salmonella-modified membranes (see ***R4****).*

Moreover, to our knowledge, we are the first to demonstrate the induction of epithelial cell differentiation as a host cell response to Salmonella infection. More specifically, cell adhesion, extracellular matrix organizations and related terms, e.g., 'collagen-containing extracellular matrix' (e.g., including collagen α-3, thrombospondin-1 and Galectin-1) (23) and 'cell adhesion mediated by integrin' (e.g., with up to over 7-fold upregulation of the integrins α-2 (ITGA2), α-5 (ITGA5), α-V and β, besides ICAM-1, ADAM9 and vitronectin)) represented upregulated processes over the time course of the infection, and this opposed to the intracellular actin cytoskeleton downregulation observed at late time points post infection. Some selected representative protein profile plots of the aforementioned significantly regulated categories are shown in previous Supplementary Figure S4, now ***Figure 3****. Jointly, these cellular and phenotypic hallmarks reflect epithelial cellular differentiation induced early upon infection (24). As such, our findings seem to indicate that cell differentiation may generate physiological heterogeneity in S. Typhimurium infected epithelial cells as previously shown for the S. Typhimurium mediated reprogramming of macrophage hosts towards alternative anti-inflammatory (i.e., M2) polarized macrophages, a pathogenic strategy enabling (a subpopulation of) intracellular bacteria to survive and reprogram their host cell to enable bacterial survival and pathogenic spread (25). As such these findings open up an interesting avenue for future research.*

Since in line with previous studies, a complex response to bacterial infection over time was observed, we now also emphasize in the discussion that e.g. strain-specific responses of the host to Salmonella infection (considering an e.g., SPI-2 mutant) could be interrogated to uncover unique profiles of pathogen/host interdependencies over the time course of the infection.

Response to Reviewers comments Part III - Minor Issues: Editorial and Data Presentation Modifications

---

Reviewer #2: Could not decide whether the above "Major" issues should be Minor, instead

R12 - Reviewer #3: Line 34 it reads "bacterial proteome response during infection progression infection by permitting quantification of low » please delete one "infection" it is double

Thank you for noticing this typo in the abstract, the sentence has been corrected.

R13 - Lines 304-308 it reads ….” Since extensive transcriptional reprogramming was shown to accompany the successful colonization of the epithelial cytosol, a niche occupied by a hyper-replicating Salmonella sub-population especially at later times post-infection, it is noteworthy that upregulation of cytosol signature genes previously reported to be transcriptionally upregulated in Gram-negative pathogens colonizing the cytosol were also observed as being regulated in our proteome study »

How did you control whether bacteria where cytosolic or vacuolar? Did you follow GFP bacteria before protein preparation or is this an assumption form the protein signature?

*While inferred from the data, examining GFP-expressing Salmonella SL1344 strain JVS-3858 (22) over the time course of infection by monitoring of GFP-positive object count and size (new **Supplementary Figure S1**), further supports this assumption. More specifically, green object counts were shown to follow a 2-step progression curve; a first steep rise in counts observed between 2 and 5 hpi, followed by a plateauing phase before another, though less steep increase in observed counts at ~8 hpi plateauing around 16-17 hpi, with the second increase indicative of the hyper-replication of cytosolic Salmonella. Further, green object size also indicates that a maximum object size was observed at ~8 hpi, indicating an accumulation of Salmonella withing the contained vacuoles until 8hpi, before vacuolar escape and more diffuse bacterial spread – as also inferred from the up to 2-fold decrease in GFP-positive object size - in the cytosol.*

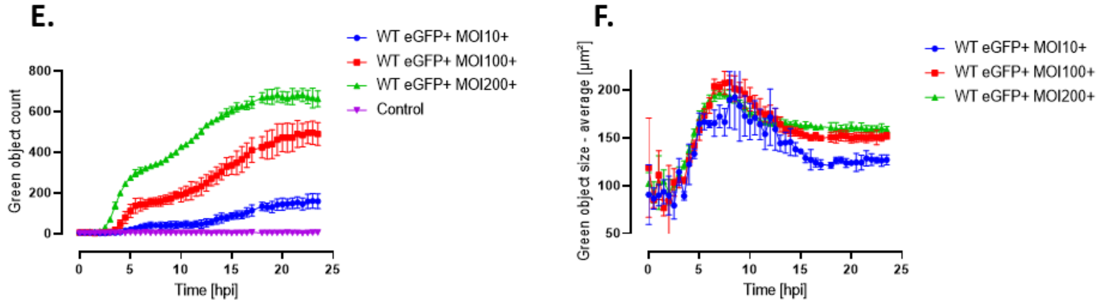

Figure S1| *Characterization of epithelial Salmonella infection model. HeLa cells (1,875 x 10^4 HeLa cells) were infected with S. Typhimurium at MOIs 10 (blue), 100 (red) or 200 (green). Using a constitutively eGFP-expressing strain of SL1344 (22) (WT eGFP) green objects were counted (E) and their average size (µm^2) determined (F). Fluorescence measurements were done using bottom reading and using an excitation wavelength of 485 nm (width 20 nm) and emission of 535 nm (width 20 nm) over the time course of the infection. Due to technical errors during measurement, the 17.5 and 18 hpi recordings were omitted.*

R14 - Figure 2: could perhaps be in the supplement, it is again very general, and no real functional conclusion can be drawn from this figure

As requested by the reviewer, we now moved the figure to the supplementary information.

R15 - Figure 3: what are the different clusters and numbers e.g. Cluster 1 (#286). In the way it is shown this figure is very general and one does not get the functional implications.

*This figure was included to give an overall impression of the observed ANOVA regulations and shows different clusters of regulation over the time course of infection (corresponding clusters of individual human proteins are given in the Supplementary Table 2C (as indicated in the corresponding figure legend) (see also comment **R7**). More specifically, clustering of proteins via hierarchical clustering highlighted protein groups associated with a specific*

temporal host response indicative that time-specific responses are induced. As subsequent figures focusses on functional data interpretation, this figure has now been moved to the Supplementary information.

R16 - I would like to see figures S4 and S5 in the main text

As suggested by the reviewer, these figures have now been moved to the main text.

References

1. Qi L, Hu M, Fu J, Liu Y, Wu M, Yu K, Liu X. 2017. Quantitative proteomic analysis of host epithelial cells infected by Salmonella enterica serovar Typhimurium. Proteomics 17.
2. Powers TR, Haeberle AL, Predeus AV, Hammarlof DL, Cundiff JA, Saldana-Ahuactzi Z, Hokamp K, Hinton JCD, Knodler LA. 2021. Intracellular niche-specific profiling reveals transcriptional adaptations required for the cytosolic lifestyle of Salmonella enterica. PLoS Pathog 17:e1009280.
3. Li Z, Liu Y, Fu J, Zhang B, Cheng S, Wu M, Wang Z, Jiang J, Chang C, Liu X. 2019. Salmonella Proteomic Profiling during Infection Distinguishes the Intracellular Environment of Host Cells. mSystems 4.
4. Vogels MW, van Balkom BW, Heck AJ, de Haan CA, Rottier PJ, Batenburg JJ, Kaloyanova DV, Helms JB. 2011. Quantitative proteomic identification of host factors involved in the Salmonella typhimurium infection cycle. Proteomics 11:4477-91.
5. Noster J, Chao TC, Sander N, Schulte M, Reuter T, Hansmeier N, Hensel M. 2019. Proteomics of intracellular Salmonella enterica reveals roles of Salmonella pathogenicity island 2 in metabolism and antioxidant defense. PLoS Pathog 15:e1007741.
6. Liu Y, Zhang Q, Hu M, Yu K, Fu J, Zhou F, Liu X. 2015. Proteomic Analyses of Intracellular Salmonella enterica Serovar Typhimurium Reveal Extensive Bacterial Adaptations to Infected Host Epithelial Cells. Infect Immun 83:2897-906.
7. Liu Y, Yu K, Zhou F, Ding T, Yang Y, Hu M, Liu X. 2017. Quantitative Proteomics Charts the Landscape of Salmonella Carbon Metabolism within Host Epithelial Cells. J Proteome Res 16:788-797.
8. Rogers LD, Brown NF, Fang Y, Pelech S, Foster LJ. 2011. Phosphoproteomic analysis of Salmonella-infected cells identifies key kinase regulators and SopB-dependent host phosphorylation events. Sci Signal 4:rs9.
9. Fels U, Gevaert K, Van Damme P. 2017. Proteogenomics in Aid of Host-Pathogen Interaction Studies: A Bacterial Perspective. Proteomes 5.
10. Liu Y, Mi Y, Mueller T, Kreibich S, Williams EG, Van Drogen A, Borel C, Frank M, Germain PL, Bludau I, Mehnert M, Seifert M, Emmenlauer M, Sorg I, Bezrukov F, Bena FS, Zhou H, Dehio C, Testa G, Saez-Rodriguez J, Antonarakis SE, Hardt WD, Aebersold R. 2019. Multi-omic measurements of heterogeneity in HeLa cells across laboratories. Nat Biotechnol 37:314-322.
11. Vorwerk S, Krieger V, Deiwick J, Hensel M, Hansmeier N. 2015. Proteomes of host cell membranes modified by intracellular activities of Salmonella enterica. Mol Cell Proteomics 14:81-92.
12. Smith AC, Heo WD, Braun V, Jiang X, Macrae C, Casanova JE, Scidmore MA, Grinstein S, Meyer T, Brumell JH. 2007. A network of Rab GTPases controls phagosome maturation and is modulated by Salmonella enterica serovar Typhimurium. J Cell Biol 176:263-8.
13. Reuter T, Vorwerk S, Liss V, Chao TC, Hensel M, Hansmeier N. 2020. Proteomic Analysis of Salmonella-modified Membranes Reveals Adaptations to Macrophage Hosts. Mol Cell Proteomics 19:900-912.
14. Spano S, Galan JE. 2018. Taking control: Hijacking of Rab GTPases by intracellular bacterial pathogens. Small GTPases 9:182-191.
15. Auweter SD, Bhavsar AP, de Hoog CL, Li Y, Chan YA, van der Heijden J, Lowden MJ, Coombes BK, Rogers LD, Stoynov N, Foster LJ, Finlay BB. 2011. Quantitative mass spectrometry catalogues Salmonella pathogenicity island-2 effectors and identifies their cognate host binding partners. J Biol Chem 286:24023-35.

16. Miao EA, Brittnacher M, Haraga A, Jeng RL, Welch MD, Miller SI. 2003. Salmonella effectors translocated across the vacuolar membrane interact with the actin cytoskeleton. Mol Microbiol 48:401-15.
17. Fijalkowski I, Willems P, Jonckheere V, Simoens L, Van Damme P. 2022. Hidden in plain sight: challenges in proteomics detection of small ORF-encoded polypeptides. microLife 3.
18. Willems P, Fels U, Staes A, Gevaert K, Van Damme P. 2021. Use of Hybrid Data-Dependent and -Independent Acquisition Spectral Libraries Empowers Dual-Proteome Profiling. J Proteome Res doi:10.1021/acs.jproteome.0c00350.
19. Searle BC, Pino LK, Egertson JD, Ting YS, Lawrence RT, MacLean BX, Villen J, MacCoss MJ. 2018. Chromatogram libraries improve peptide detection and quantification by data independent acquisition mass spectrometry. Nat Commun 9:5128.
20. Hannemann S, Gao B, Galan JE. 2013. Salmonella modulation of host cell gene expression promotes its intracellular growth. PLoS Pathog 9:e1003668.
21. Cox J, Mann M. 2012. 1D and 2D annotation enrichment: a statistical method integrating quantitative proteomics with complementary high-throughput data. BMC Bioinformatics 13 Suppl 16:S12.
22. Hautefort I, Proenca MJ, Hinton JC. 2003. Single-copy green fluorescent protein gene fusions allow accurate measurement of Salmonella gene expression in vitro and during infection of mammalian cells. Appl Environ Microbiol 69:7480-91.
23. Naba A, Clauser KR, Hoersch S, Liu H, Carr SA, Hynes RO. 2012. The matrisome: in silico definition and in vivo characterization by proteomics of normal and tumor extracellular matrices. Mol Cell Proteomics 11:M111 014647.
24. Gomez-Lamarca MJ, Cobreros-Reguera L, Ibanez-Jimenez B, Palacios IM, Martin-Bermudo MD. 2014. Integrins regulate epithelial cell differentiation by modulating Notch activity. J Cell Sci 127:4667-78.
25. Majstorovic A, Hill PWS, Helaine S. 2020. Salmonella antibiotic persistence and macrophage polarisation. Access Microbiology 2.
